# Exploratory ensemble designs for environmental models using k-extended Latin Hypercubes

**DOI:** 10.1002/env.2335

**Published:** 2015-03-24

**Authors:** D Williamson

**Affiliations:** College of Engineering, Mathematics and Physical Sciences, University of ExeterExeter, U.K.

**Keywords:** climate models, diagnostics, emulation, initial condition uncertainty, uncertainty quantification

## Abstract

In this paper we present a novel, flexible, and multi-purpose class of designs for initial exploration of the parameter spaces of computer models, such as those used to study many features of the environment. The idea applies existing technology aimed at expanding a Latin Hypercube (LHC) in order to generate initial LHC designs that are composed of many smaller LHCs. The resulting design and its component parts are designed so that each is approximately orthogonal and maximises a measure of coverage of the parameter space. Designs of the type advocated for in this paper are particularly useful when we want to simultaneously quantify parametric uncertainty and any uncertainty due to the initial conditions, boundary conditions, or forcing functions required to run the model. This makes the class of designs particularly suited to environmental models, such as climate models that contain all of these features. The proposed designs are particularly suited to initial exploratory ensembles whose goal is to guide the design of further ensembles aimed at, for example, calibrating the model. We introduce a new emulator diagnostic that exploits the structure of the advocated ensemble designs and allows for the assessment of structural weaknesses in the statistical modelling. We provide illustrations of the method through a simple example and describe a 400 member ensemble of the Nucleus for European Modelling of the Ocean (NEMO) ocean model designed using the method. We build an emulator for NEMO using the created design to illustrate the use of our emulator diagnostic test. © 2015 The Authors. *Environmetrics* published by John Wiley & Sons Ltd.

## 1. Introduction

The use of complex mathematical models, typically in the form of coupled ordinary, partial or stochastic differential equations, to describe complex physical systems is important in many diverse scientific disciplines. Where these equations cannot be solved analytically and must, instead, be solved numerically using computer simulations, the developed models are referred to in the statistics literature as ‘computer models.’ Such models are ubiquitous in environmental applications, with examples including models used to study the risk and impacts of natural hazards such as volcano eruptions and tsunamis, and models used to study the past, the present and the future behaviour of the Earth's climate. The study of complex systems, such as the climate, using computer models introduces a number of sources of uncertainty that must be quantified so that appropriate inferences about the system can be made and to facilitate decision making. Quantifying this uncertainty through the design and analysis of computer experiments has become an active and important avenue of statistical research, and a rich methodology for addressing typical problems now exists (Santner *et al.*, 2003).

That methodology is based on *emulators*, sometimes referred to as ‘surrogate models’. An emulator is a stochastic representation of the computer code that, for any setting of the inputs to the model (what we will also refer to as the model parameters), returns a prediction for the model response and an uncertainty on that prediction. This is powerful because an emulator can produce a prediction in a fraction of the time required to run the computer model. Computer models can take anywhere from a few seconds to a few months to evaluate for any particular choice of the inputs, so an emulator, once built, becomes an invaluable tool for exploring the parameter space, calibrating the model and providing decision support. There are now published statistical methodologies using emulators to assist in each of these problems (see, for example, Oakley & O'Hagan, 2004; Lee *et al.*, 2011; Kennedy & O'Hagan, 2001; Vernon *et al.*, 2010; Williamson & Goldstein, 2012).

An emulator is built by first systematically sampling the input parameter space to design a set of ‘training runs’, typically referred to as an ‘ensemble’ in the environmental literature, and using the ensemble to fit the emulator. The central role played by the emulator has lead to a large literature on designs aimed at building accurate emulators. Before any runs of the model have been observed, this design is most likely to take the form of a ‘space filling’ design: a design aimed at exploring as much of the parameter space as possible. Many classes of space filling designs have been proposed, including Sobol sequences (Fang *et al.*, 2005), Orthogonal arrays (Tang, 1993) and Latin Hypercubes (LHCs) (McKay *et al.*, 1979), with the latter being the most popular in practice.

A LHC of size *n* divides each dimension of the input space into *n* bins and ensures exactly one point is sampled from each bin. The sampling can be done with respect to any prior distribution on the parameters, but in a purely exploratory design, such as those we are discussing in this paper, it is most common to use a uniform LHC, whereby the range of each input is divided into *n* equally spaced intervals and the resulting LHC represents each interval exactly once. There is now a considerable literature on space filling LHC designs focused on constructing designs so that they satisfy one or a number of desirable properties. For example, they can be designed to have a specified correlation structure (Iman & Conover, 1982), to be orthogonal or nearly orthogonal (Sun *et al.*, 2009; Gu & Yang, 2013), or to maximise some measure of coverage such as the minimum distance between points as in the case of maximin LHCs (Morris & Mitchell, 1995).

The popularity of LHCs has led to research into extending initial LHC designs in a way that retains the properties of the original. Indeed, the interest in Sobol sequence designs was due to the ability to add to the design once created (Challenor, 2011). The rationale is that, having spent an initial budget of model runs on an *n*-point LHC, we may get access to further runs and would like to know where to put them. Sallaberry *et al.* (2008) introduced a method for doubling the size of a LHC whilst retaining approximately the same rank correlation structure. They provide an algorithm for extending a LHC of size *m* into one of size 2*m* and state that the algorithm could be generalised to extend to any size *km*, with *k*, a positive integer. In this paper we will provide this generalisation.

It is the view of this author that, having run an initial exploratory ensemble using a space filling design, an optimal ‘second wave’ ensemble design is unlikely to take the form of a LHC or even be space filling, and that information from the initial ensemble should be used to guide the analyst towards regions of interest that may be more heavily sampled, and away from uninformative or unphysical regions of space, (see, for example, Loeppky *et al.*, 2009). For example, suppose an initial ensemble of climate model runs allowed us to confirm that half of our parameter space contained only ice planets. We would be very reluctant to spend about half of our remaining run budget exploring this region of parameter space in a second ensemble as we would if our design was a LHC in the full space, because we are interested in the behaviour of the model when the global temperature is not completely unphysical. However, the ability to expand space filling designs may be extremely useful in the construction of the initial design itself.

There are a number of reasons we might wish to do this. One such reason may be found in the desire to construct robust emulators of the model. Typically, an emulator is built using part, or all, of an ensemble and then validated using a number of diagnostic checks (Bastos & O'Hagan, 2009). Some of the more powerful tests involve assessing the predictive performance of the emulator on as yet unseen runs. If subsets of the design have similar properties to the whole, for example if they are also LHCs, these can be left out and used in validation and diagnostic checking that aim to assess performance throughout parameter space.

Perhaps, the best case for designs of the type advocated here is for computer models whose output depends on uncertain initial conditions, forcings or boundary conditions. Environmental models are typically of this variety and, indeed, climate models have all three properties. For a climate model, the initial conditions represent the temperature, salinity and momentum of air/water for every grid box in the model at the time any parameters are changed, or any forcings (e.g. increasing CO2 or aerosols) are applied, and boundary conditions, for example, might tell the model what to do with water at the poles. Initial condition uncertainty (and the other sources) must be addressed as part of the emulation and analysis of the model output. To quantify initial condition (IC) uncertainty, for example, part of the ensemble requires no change to the parameters whilst the initial conditions are varied. If the IC uncertainty does not depend on the parameters, the ‘initial condition ensemble’ can be run in isolation, merely factoring into budget constraints. However, in our experience, this is not the case, and the sensitivity to the ICs depends on where you are in parameter space. If our master space filling design is constructed using smaller space filling designs with the same property, we can select one of these sub-designs and vary the initial conditions for each member of it, This enables us to model the IC uncertainty as a function of the parameters by emulating the variance as a first step to emulating the full model, (see Vernon and Goldstein, [Bibr b36],[Bibr b37] for examples emulating the variance then the mean in systems biology).

Sliced Latin Hypercubes (Qian, 2012; Huang *et al.*, 2014; Yin *et al.*, 2014; Ai *et al.*, 2013) are LHCs that are constructed using a set of smaller LHCs. These designs are most similar to the ones we introduce in this paper. Indeed, k-extended LHCs may be seen as a special case of sliced LHCs where instead of the design generation algorithm focussing on setting all smaller sub-LHCs and the main together (in order to optimise some criterion), ours are ‘grown’ from a master LHC. Although sliced LHCs would allow us to achieve better emulator diagnostics (as described later) than a standard LHC (just as the method we will advocate here would), the existence of an important sub-LHC from which the design is ‘grown’ will be more useful for exploring IC uncertainty.

Nested LHCs (Qian, 2009) are LHC designs that contain a sub-design that is also a LHC. They are motivated by computer experiments for hierarchies of computer models of different fidelity or resolution. Many expensive models can take so long to run, that only a handful of runs can be performed. However, low resolution versions often exist that can be used to assist in emulation of the high-resolution version. The idea is that a large design is used to emulate the low resolution model, and then this information plus a small design on the high resolution model is used to emulate the model of interest (see, for example, Williamson *et al.*, 2012). Nested LHCs are one type of design aimed at simultaneously designing for both of these experiments at the same time. We might view the designs advocated in this paper as containing useful features of both nested and sliced LHCs.

In this paper, we generalise the algorithm of Sallaberry *et al.* (2008) to the case where 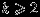, in order to construct initial LHC designs that are made up of many LHCs. We term our designs ‘k-extended LHCs’. We also present an algorithm for ensuring that sub-designs and the whole design are ‘orthogonal-maximin’ designs using a definition similar to that used by Joseph & Hung (2008). We discuss exploring IC uncertainty and emulator diagnostics with k-extended LHCs and introduce a new form of diagnostic available for ensembles generated from k-extended LH designs. We apply the technology to the design of an exploratory ensemble of the Nucleus for European Modelling of the Ocean (NEMO) ocean model (Madec, 2008) at 2^*o*^ resolution, and we use the ensemble to emulate the model and validate our emulator. We also provide R-code in the Supporting Information that can be used to generate designs of the type described in the paper.

In Section 2, we formally define LHCs and present the k-extension algorithm. In Section 6, we introduce an algorithm for generating orthogonal-maximin k-extended LHCs and compare these designs with sliced LHCs in a simple numerical example. In Section 8, we argue for the application of our approach to quantifying initial condition uncertainty in certain applications and present a novel emulator diagnostic available for ensembles with these designs. Section 11 describes the design of our ocean model ensemble and illustrates the use of our new emulator diagnostic. Section 16 contains discussion. A technical appendix contains two results and proofs required by the algorithm of Section 6. The R code implementing the design algorithm is part of the Supporting Information.

## 2. Latin Hypercubes and K-Extensions

### 2.1. Latin Hypercubes

Let *f*(*x*) denote a computer model with input vector **x** = (*x*_1_,…,*x*_*m*_)^*T*^, with **x** in a continuous, predefined real space 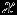 that can be scaled to the unit hypercube [0,1]^*m*^. Throughout this paper, we assume that nothing is known about *f*, other than the parameter ranges that define 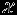, so that, as part of an initial exploratory design, we do not favour points in any particular regions of 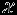 by imposing probability distributions on each of the *m* inputs.

Let *r*_*j*_ denote the range of *x*_*j*_ and suppose that each *r*_*j*_ is divided into *n* equal intervals *r*_*ij*_,*i* = 1,…,*n*, then an *n*-point LHC, *X* = (**x**_1_,…,**x**_*n*_)^*T*^, such that there is a unique row of *X*, **x**_*k*_, with *x*_*kj*_∈*r*_*ij*_ for *j* = 1,…,*m* and *i* = 1,…,*n*. In words, each sub-interval *r*_*ij*_ is represented exactly once in the LHC, *X*.

A LHC is easily generated by first selecting an *r*_*ij*_ for *j* = 1,…,*m*, and then uniformly choosing a point from the identified rectangular solid 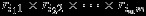 (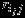 is a particular sub-interval chosen for variable *j*, so at this first step, each *i*_*j*_∈{1,…,*n*}). This obtains **x**_1_. Remove the selected 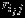 from the set of available sub-intervals {*r*_1*j*_,…,*r*_*nj*_} for *j* = 1,…,*m*, then repeat the procedure for identifying a solid, obtaining a new point **x**_2_, reducing the set of sub-intervals and so on until **x**_1_,…,**x**_*n*_ have been generated. There are statistical software packages such as lhs in R, that have been developed to do this.

This procedure can be used to generate a LHC, but it is no guarantee of generating a good one with desirable space filling properties. For example, there is no reason that the resulting design should not have points close together or be highly correlated. ‘Optimal’ LHC algorithms attempt to generate a LHC with desirable properties. For example, a maximin LHC seeks to maximise the minimum distance between points. This can be done by generating a random LHC using the aforementioned procedure then permuting the entries in each column in order to optimise some pre-chosen criterion. The function maximin in the lhs package in R performs this function in order to maximise the minimum distance between points.

A useful way to think about generating LHCs for us will be to consider integer LHCs, whereby the *n* × *m* LHC has each column filled by some permutation of the integers {1,…, n}. The *i*-th row of this matrix, (*i*_1_,…,*i*_*m*_) with *i*_*k*_∈{1,…,*n*}, identifies the ranges of sub-intervals 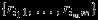, pointing to a solid, 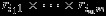, from which **x**_*i*_ can be chosen as described previously. We can easily permute the entries of any individual column or of multiple columns in order to generate new LHCs. The integer representation of the LHC, *X*, can be viewed as a rank representation of the design, with the rank of each input *k* in the *j*-th design point being the *jk*-th entry of the integer LHC used to generate *X*. This idea is central to extension of LHCs proposed by Sallaberry *et al.* (2008), which we will describe in more generality here. It will also be useful to us in optimising our LHC and its component parts.

### 2.2. K-extended Latin Hypercubes

We now present our generalisation of the extension algorithm of Sallaberry *et al.* (2008). Although Sallaberry *et al.* (2008) describe the algorithm for extending an *n*-point LHC into a 2*n* point LHC and state that it can be generalised to extension to *kn* point LHCs with positive integer *k*, we present the algorithm for *kn* points directly. We do this because firstly, the extension to the general case is not trivial, and secondly, because our goal is to produce *initial* designs that are LHCs themselves made up of many component LHCs with the whole and component parts satisfying desirable properties. In particular, we will want to maximise some measure of coverage, as will be described in Section 6, and produce a design where each variable is as orthogonal as possible. The reasoning behind the latter design goal is to ensure identifiability of main parameter effects during emulation. Our ultimate aim is to use the design to build a robust emulator that can then be used in any given uncertainty quantification method or in order to guide future designs to regions of the parameter space that are important.

**Figure 1:**
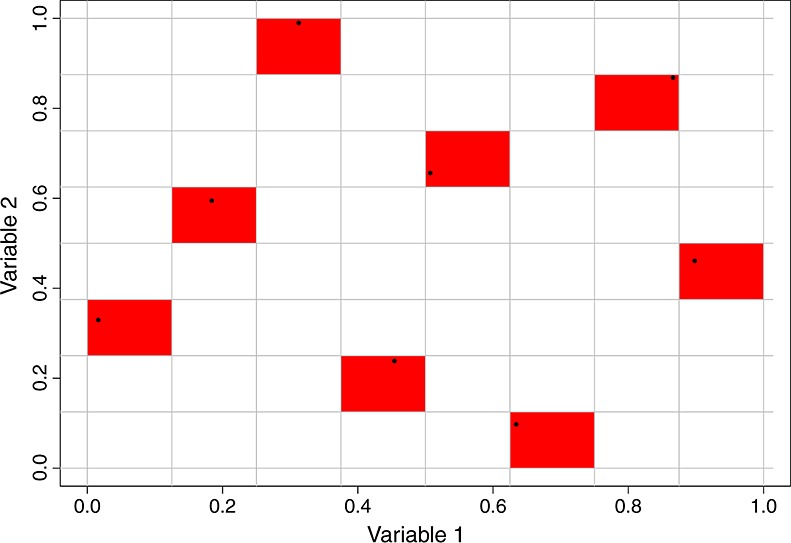
An eight-point Latin Hypercubes (LHC) in two dimensions generated via an integer LHC. The shaded boxes highlight the solids identified by the integer LHC, with the points representing the LHC in [0,1]^2^.

What follows is a description and justification of the procedure followed by a technical statement of the algorithm. We start by generating an *n*-point LHC, starting with an integer version and selecting the actual values in *X* as described in the previous section. We ensure that our initial design has desirable properties (e.g. coverage) at this stage by ensuring our design optimises some pre-chosen criterion, as described in Section 6. An example eight-point LHC in two dimensions is shown in Figure 1, with the points representing the rows of *X* and the shaded boxes representing the rows of the integer LHC chosen to generate the points. For example, the left most shaded region represents the row of the integer LHC with entries {1,3}.

**Figure 2:**
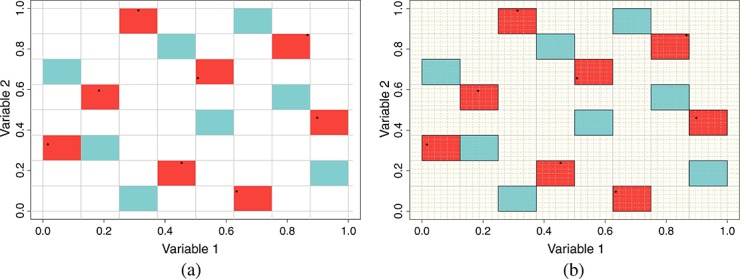
(a) The integer Latin Hypercube (LHC) used to generate the first extension of our example eight-point LHC in two dimensions. The cyan panels highlight the new integer LHC that will be used to generate new points. (b) Dividing each solid in the identified integer LHC into *k*^*m*^ equally sized solids. One of the mini-solids that has no points along it in either dimension will be chosen at random to contain the new LHC points for each of the larger cyan solids identified by the integer LHC

Our goal is to end up with a *kn*-point LHC comprising *kn*-point LHCs, so we require a further *k* − 1 extensions to this LHC. For the first extension, we choose another integer LHC so that both, it and the 2*n* × *m* matrix of integers, formed by stacking the two integer LHCs row-wise meets our criteria described in Section 6. This second integer LHC identifies the *m*-dimensional rectangular solids in which the new points will reside, as depicted for our ongoing example in Figure 2(a). We now divide each of the identified solids into *k*^*m*^ equally sized solids by dividing the range of each input within that solid into *k* identically sized bins, as shown in Figure 2(b).

For each sub-solid within each solid specified by our new integer LHC, we can search each dimension of the current LHC, *X*, for a point anywhere in the design within the range of the dimension in question determined by the chosen sub-solid. One can view this exercise as looking through our chosen sub-solid along each of its dimensions to see if any existing design points are visible. Note that (*k* − 1)^*m*^ of these sub-solids will have no visible points along any of the dimensions. Select one of these sub-solids at random and uniformly select a point within it. Repeat this for each solid identified by the integer LHC and extend the design *X* by adding the *n* new rows identified by this procedure. Note *X* is no longer a LHC, but it is a design comprising 2 *n*-point LHCs.

**Figure 3:**
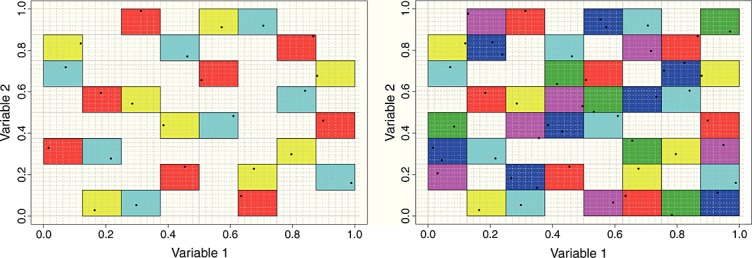
(a) The first three 8-point Latin Hypercubes (LHCs) generated during application of the extension algorithm with different coloured grid squares representing the three integer LHCs used to identify the regions of the new points. (b) The full 40 point LHC comprising five 8-point LHCs. The integer LHCs used to generate each extension are highlighted as different coloured grid squares.

We now repeat this process a further (*k* − 2) times. First is choosing an *n*-point integer LHC with desirable properties when combined with all other integer LHCs used in the design, then dividing each identified solid into *k*^*m*^ identically sized solids before looking along each dimension of the design through each sub-solid in order to identify all sub-solids with no visible points along any dimensions. When adding the *j*-th additional *n*-member LHC to the original *X*, there will be (*k* − *j*)^*m*^ such solids from which a sub-solid may be selected at random and a design point selected uniformly from within. This is shown for a five-extended LHC of dimension 2 and size 40 in Figure 3. Figure 3(a) shows the process after two extensions with the three chosen integer LHCs highlighting the selected solids coloured in red, cyan and yellow, and the chosen points in black. Note that along any of the sub-rows/columns, there is a maximum of one point. When the final extension occurs, exactly one sub-solid in each identified solid will be eligible for a new point, and following its placement and addition of all points into *X*, every one of the *kn* equally sized sub-intervals of each dimension of 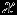 will be represented exactly once in *X*. So *X* is a *kn* LHC composed of *kn*-point LHCs where the whole and the sequentially generated sub-designs have been engineered to have desirable properties. This is depicted for our on-going example in Figure 3(b).

### 2.3. K-extended Latin Hypercube generation algorithm

**Step 1** Choose an *n*-point integer LHC with optimal properties (Section 6) and use the procedure defined in Section 2.1 to generate an *n*-point LHC, *X*, of points in 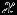. Set counter *c* = 2.

**Step 2** Choose a new *n*-point integer LHC with optimal properties (Section 6) when stacked by row with the previous *c* − 1integer LHCs used in the procedure.Let *s*_*i*_ represent rectangular solid *i*, *i* = 1,…,*n*, represented by the *i*-th row of the new integer LHC, 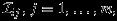 where 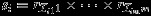.

**Step 3** For each *i* = 1,…,*n*, divide *s*_*i*_ into *k*^*m*^ identically sized sub-solids by dividing each edge 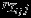 of *s*_*i*_ into *k* equally spaced intervals 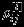 for *l* = 1,…,*k*;*j* = 1,…,*m*; and with each sub-solid of the form


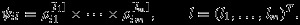


**Step 4** For each *i* = 1,…,*n*, identify the (*k* − *c* + 1)^*m*^ solids *ψ*_*ił*_ such that 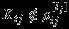 for *t* = 1,…,(*c* − 1)*n* and *j* = 1,…,*m*, and choose one at random. Label this choice 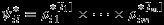.

**Step 5** Define the matrix 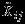 by uniformly sampling from 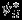. Specifically, by sampling uniformly from each of the intervals 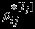 for *j* = 1,…,*m*.

**Step 6** Let


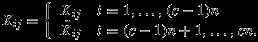


Let *c* = *c* + 1, if *c* > *k* STOP, else go back to Step 2.

## 3. Generating Orthogonal Maximin K-Extended Latin Hypercubes

It is the goal of this paper to advocate for k-extended LHCs as a good initial exploratory design capable of allowing us to build and validate an emulator that can then be used by the analyst, perhaps in further interrogation of the model. Hence, our two main criteria will be that the design is ‘space filling’ so that we explore as much of the parameter space as possible, and also that it is uncorrelated, so that the main effects, at least, are identifiable during the statistical modelling phase.

To achieve this, we use the idea of finding what Joseph & Hung (2008) termed ‘orthogonal-maximin LHCs’, defined to minimise a weighted sum of measures of total correlation in the design and coverage on an appropriate scale. We work with *M*^*c*^, the integer representation of the design at Step 2 of the k-extension algorithm, where column *j* of 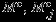, has c permutations of the integers {1,…,*n*} for *j* = 1,…,*m*. The correlation component of the Joseph & Hung (2008) criterion is the average square correlation on the lower triangle of the design's correlation matrix:


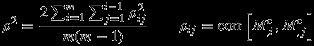


Coverage is measured using the *φ*_*p*_ statistic


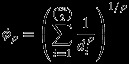


with 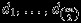 the inter-site distances in 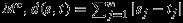. This is the same criterion commonly minimised in the design literature when looking for maximin designs as it can be shown that as *p* gets high, minimising *φ*_*p*_ is equivalent to finding the minimum *d*_*i*_ and maximising it (Morris & Mitchell, 1995).

Joseph & Hung (2008) ensure that the two criterea are on the same scale by deriving upper and lower bounds *φ*_*p*,*U*_ and *φ*_*p*,*L*_ for *φ*_*p*_, and then their criterion is


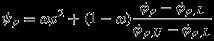
(1)

for weight *ω* that can be chosen by the analyst to represent their preferences for orthogonality or coverage. We will use a similar criterion to theirs to optimise each phase of our k-extension. However, we need a slightly different version of *φ*_*p*_ to assess the coverage of the designs formed at Step 6 of the algorithm and this leads to different bounds *φ*_*p*,*L*_ and *φ*_*p*,*U*_.

We define


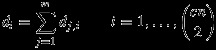


where the *d*_*j*,*i*_ are inter-site distances for column *j* of *M*^*c*^. Because we are extending an original LHC, each column of *M*^*c*^ will have *c* repeats of the integers {1,…,*n*}. This means that some of the *d*_*j*,*i*_s as defined previously, are zero, which leads to a theoretical upper bound *φ*_*p*,*U*_ of *∞*. To overcome this, we set any zero *d*_*j*,*i*_ to 1/*k*, the smallest distance between the centres of the sub-solids that the k-extended points will eventually occupy. With this modification to *φ*_*p*_, we can show that


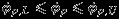


with


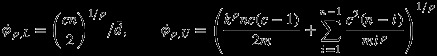


and


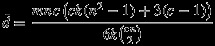


A derivation of these bounds is given in the Appendix. With the criterion *ψ*_*p*_ as in (1) in place, we can use a simulated annealing algorithm to ensure each extension to the design at Step 2 minimises *ψ*_*p*_. This is a common approach to optimising a criterion in computer experiment design and was proposed by Morris & Mitchell (1995). The idea for general LHCs is to choose a random column and to permute two of the entries (the result is still a LHC). If the criterion improves, the new LHC is accepted, else it is accepted with probability exp{−(*ψ*_*p*,new_−*ψ*_*p*,old_)/*t*}, where *t* is a pre-chosen ‘temperature’. A simulated annealing algorithm will repeat this many times, whilst slowly reducing *t*, until it has converged on the design with the optimum *ψ*_*p*_.

We take this same approach, but as we are extending the LHC sequentially, we preserve the first (*c* − 1)*n* rows of *M*^*c*^ and only allow elements of the last *n* rows of the candidate design to be permuted within randomly selected columns. Aside from this modification to which rows may be permuted, the algorithm we use is that of established by Morris & Mitchell (1995) as applied to *ψ*_*p*_ (also described in; Joseph & Hung, 2008), hence we do not reproduce it here.

A particular advantage of our approach to sequentially extending a LHC over a sliced LHC is that each of the designs corresponding to the first *n*,2*n*,…,*kn* points is optimal with respect to *ψ*_*p*_ (given the previous sub-designs) so can be considered an orthogonal-maximin design (although only the first and the last of these are LHCs). This can be useful, for example, for experiments that have to be queued on supercomputers where, for whatever reason, it is possible that not every run will be completed. This is not uncommon in these situations. For example, if there is a deadline, run time of the model plus the supercomputers workload and the priorities of your job on the system may mean that only the first *N*, say (with *N* < *kn*) are completed in time. Our design construction would ensure that there was a sub-design of *ln*(*l* < *k*) runs that were optimally orthogonal and space filling (via *ψ*_*p*_ and given the previous (*l* − 1) sub-designs) consisting of *ln*-member LHCs. The criterion we present here is implemented in the R-code that accompanies the paper.

Before proceeding to compare the performance of orthogonal-maximin k-extended LHCs with other designs, we note here that our k-extension algorithm, when finding an orthogonal-maximin k-extended LHC, only optimises our criterion for the integer LHCs that make up the design. Steps 4 and 5 of our procedure put the extended points in random available sub-regions of the solids identified by our integer design. A future extension of this methodology might look at selecting optimal sub-region location for the extra points.

### 3.1. Numerical comparison of a k-extended LHC and a sliced LHC

We illustrate the differences between sliced LHCs and k-extended LHCs through a short numerical example. The R package SLHD generates maximin sliced LHCs. Our comparison makes use of sliced LHCs generated by this package, and maximin LHCs generated by the maximinLHS function in the package lhs. Both packages are freely available online from CRAN. We compare these with performance of k-extended orthogonal-maximin LHCs generated using the aforementioned algorithm and optimising *ψ*_*p*_ with *p* = 50 and *ω* = 0.2 reflecting a leaning towards obtaining space filling designs over orthogonality.

**Figure 4:**
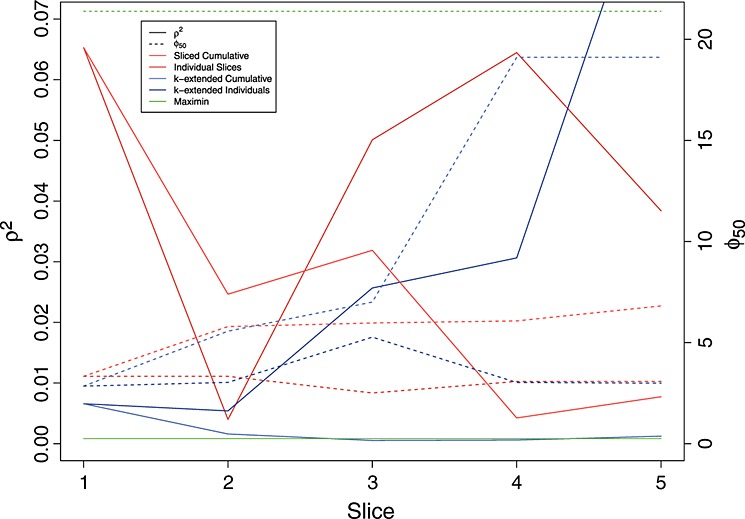
Comparing orthogonality and coverage of a sliced Latin Hypercubes (LHC), a k-extended LHC and a maximin LHC. Red lines show the sliced LHC performance, blue the k-extended LHC and green the maximin LHC. Darker colours represent the values of each measure for each individual sub-LHC (hence, there are no dark green lines) and lighter colours represent the cumulative values of each measure using the first 8, 16, 24, 32 and 40 points in the design. Solid lines represent *ρ*^2^ and dashed lines are *φ*_50_ both calculated using the scaled design values.

For both tests, we compare a full LHC of size 40 with 2 columns on [0,1], made with five and eight member LHCs (as in our earlier example). The maximin LHC will be 40 × 2 and will not be composed of five smaller LHCs as with our other designs. Figure 4 compares the values of *ρ*^2^ (solid lines) and *φ*_50_ (dashed lines) calculated on the scaled representation of the designs. Blue lines represent the k-extended LHC, red the sliced LHC and green the maximin LHC. Darker lines in each colour represent the values of the statistics for the individual sub-LHC's and lighter lines represent the cumulative values calculated using the first slice, the first two combined and so on. As the maximin LHC is not made up of individual sub-LHCs, only the values for the full design are plotted.

Focussing first on coverage measured by *φ*_50_, we note that the maximin sliced LHC with a value of around 6.8 outperforms the k-extended LHC (19.1) and the maximin LHC (21.2) on the full LHC. Although it is expected that the sliced LHC should outperform the k-extended LHC here as it has been set up to optimise this statistic in particular, it is strange that the maximin LHC should be beaten at all. Further investigation of *φ*_50_ for 40 × 2 LHCs generated by maximinLHS gave a sampling distribution containing values as small as 9.1 and as high as 45, although values for the other two algorithms remained consistent around those generated here. As both the k-extended LHC and the sliced LHC are valid designs for maximinLHS, we can conclude that this is probably an issue with the internal optimiser used for this function.

Although the sliced LHC provides better coverage for the higher LHC, the k-extended LHC has better coverage for the first LHC and the composite of the first and second, and has similar coverage properties for the fourth and fifth sub-LHCs. Although not guaranteed, because the k-extended LHC is optimising a weighted sum of the correlation and the coverage, as we have a larger weight on coverage, we should expect the k-extended LHC to outperform the sliced LHC for the first LHC at least (and perhaps number of composites). Whilst the sliced LHC is maximin for the whole design, the k-extended LHC is ‘optimal’ (w.r.t. *ψ*_50_) for the first sub-design, then for the first 2, given the first, then for the first 3 given the first 2 and so on. The importance placed on the first sub-LHC is particularly appropriate in situations where the first design will be used to quantify any parametric dependence on initial condition uncertainty (see Section 8 for detailed discussion).

The k-extended LHC outperforms the maximin-sliced LHC across the board for *ρ*^2^, although, as correlation is not accounted for by the sliced LHC generation method, this should not be surprising. We note, in particular, the low correlation for the first LHC in the k-extended LHC. As with the results on coverage, a key advantage of using k-extended LHCs is that the first design is optimised and so can be used as a lynch pin for the design (as would be the case for a nested LHC). We discuss this further in the succeeding sections.

## 4. Exploratory designs with k-extended LHCs

In this section, we discuss two important ways in which the features of this type of exploratory design can be exploited in applications: the quantification of initial condition uncertainty and the generation of novel emulator diagnostics.

### 4.1. Initial condition uncertainty and stochastic models

In many applications, the initial conditions at which the model ought to be run are uncertain, and that uncertainty propagates to the output. This is particularly true for models that are what Williamson & Blaker (2014) referred to as ‘structurally chaotic’. A structurally chaotic model is deterministic, meaning that running the model twice for the same set of inputs and initial conditions will lead to exactly the same output. However, slight changes to either can have a substantial effect on the evolution of any time series output, Although this effect would be ‘averaged out’ if taking a long-term mean. This has also been referred to as ‘sensitive dependence on initial conditions’ (Rougier, 2013).

This is the case with climate and other environmental models, where although long-term trends and averages would generally be preserved by a slight change to the initial conditions, the evolution of the time series and the ‘weather’ at any time point may be quite different. If the goal of an analysis with a climate model is to infer something about the climate response to short-term forcing, such as that which may be expected in the first half of the 21st century, then the model's initial conditions may have a non-negligible effect on the model output. Hence, the uncertainty in the computer model response due to the initial conditions should be quantified. In the climate literature, this source of uncertainty is generally referred to as internal variability.

The contribution of this source of uncertainty need not be constant throughout parameter space. Whilst it may be natural to start with an emulator of the form


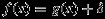
(2)

where *g*(**x**)represents the signal that might be emulated normally via a Gaussian process (see, for example Sacks *et al.*, 1989; Haylock & O'Hagan, 1996), and noise process *δ* ∼ N(0,*σ*^2^) (normality is used for illustration here rather than being crucial to the argument), it is more reasonable to expect


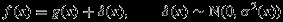
(3)

where the variance of the noise process depends on the model parameters in some way.

In order to quantify initial condition uncertainty under (2), we require repeated runs in input space where initial conditions are changed, (see Deser *et al.*, 2012, for an example of this approach applied to a climate model). The ‘initial condition ensemble’, as it is referred to in the climate literature, could then be used to estimate *σ*. However, under (3), our goal should be to model *σ* as a function of **x**, thus describing how initial condition uncertainty varies with the parameters. We then require a design in 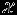 at which to vary the initial conditions. As this design will be used to emulate *σ*(**x**) in the same way, we would emulate any computer model and any properties we consider important to preserve in our design for the computer model (in this paper, we have focussed on orthogonality and good coverage) will also be desirable to preserve in this design.

As part of an initial exploratory design then, it seems natural to want to spend some of the runs on quantifying *σ*(**x**) and some modelling *g*(**x**). A k-extended LHC is a natural way of simultaneously achieving both goals. We have a LHC design that can be used to get *g*(**x**) and a small part of that design is also a separate LHC that we can use to run an initial condition ensemble. This argument also applies to stochastic simulators, where running the model at the same choice of the parameters would lead to different answers. In applications with these simulators, it is already a practice to emulate the variance as a function of the parameters (Vernon and Goldstein, [Bibr b36],[Bibr b37]), although designs for these simulators have tended to use a number of repeats for every explored parameter choice. K-extended LHCs would provide an alternative that allows the budget of runs to be more flexibly divided between the goals of modelling *σ*(**x**) and *g*(**x**).

We note that, as described previously, a nested LHC is as useful, if not equivalent to the k-extended LHC in terms of simultaneously exploring parameter and initial condition uncertainty. However, the k-extended form has more flexibility, allowing us, for example, to devote two of our sub-LHCs to initial condition ensembles so that we have an initial condition ensemble for training and one for validation. Before moving on, it is worth noting that we have not commented on how the run budget should be divided between changes to the initial conditions and to the parameters. This would provide an interesting avenue of further research. However, we suspect that the answer to this may be problem specific and that an initial exploratory design aimed at gaining enough information to answer this question may be required anyway.

### 4.2. Emulator diagnostics

The exploratory designs we advocate for here are intended to be used to build emulators, which may then be used for whatever analysis is deemed important, including the designing of subsequent runs in regions of the parameter space of interest that are identified by the emulator. For example, history matching, a statistical method that uses emulators to rule out regions of input space that lead to unphysical models (Craig *et al.*, 1996; Vernon *et al.*, 2010), points to a region of input space that may be explored by further experiment. Other subsequent designs for the computer model may be used to reduce emulator uncertainty (Loeppky *et al.*, 2009), search for an optimal parameter setting (Gramacy & Lee, 2011), or reduce uncertainty in a specific model-based forecast (Craig *et al.*, 2001).

Of importance to any of the aforementioned methods, or indeed, any analysis using an emulator in place of a computer model, is the accuracy of the emulator. Namely, that the predictions made by the emulator and the uncertainty on those predictions is consistent with untested model output. Validation and diagnostic checking aim to assess the quality of an emulator.

Suppose we are building an emulator to address the response to the model inputs only, the function *g*(**x**) in Equations (2) and (3). A typical emulator for output *i* of *g*(**x**) is then


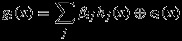
(4)

where *h*(**x**) is a vector of basis functions in **x**,*ε*_*i*_(**x**) is a weakly stationary Gaussian process and the symbol ⊕ indicates the addition of independent terms. Emulation involves selecting *h*(**x**), then fitting matrix *β* and the elements of a covariance function for *ε*(**x**), perhaps using kriging or Bayesian methods (see, for example, Santner *et al.*, 2003; Haylock & O'Hagan, 1996; Lee *et al.*, 2011).

There are a number of methods available for performing diagnostic checking of the emulator fit. For an overview, see Bastos & O'Hagan (2009). One powerful method involves reserving an input from the design, fitting the emulator using the chosen *h*(**x**) and covariance parameters, then predicting the reserved point. Doing this for each point and plotting the results is known as a ‘leave one out diagnostic plot’. By observing how often the true points lie within the relevant confidence or credible intervals specified by the emulator, the quality of the fit can be judged.

Leaving out subsets of the design is another version of this diagnostic and is one that allows the analyst to explore the sensitivity of any prior choices (such as those in *h*(**x**)) to the chosen subset. Ideally, however, these subsets would not all be clustered in one region of the input space as this might mislead the fitting of *h*(**x**) and convince the analyst that a design that really was robust was flawed based on a test of a non-balanced subset of it.

K-extended LHC designs lend themselves naturally to this type of diagnostic checking, as the *kn* member design actually has *k* ‘space filling’ sub-designs contained within it. Each sub-design may be reserved from the whole and the emulator fit with the remaining points. Note that the remaining points do not form a LHC, but are still well spread in the input space because they comprise (*k* − 1) space filling LHCs of size *n*. The reserved points can be predicted using the emulator built without them and the predictions compared with the true values. We now have *n* prediction errors corresponding to a LHC (the reserved sub-design) in the inputs. We term this diagnostic a Leave One Latin Hypercube Out (LOLHO) diagnostic and it is available for k-extended and sliced LHCs.

We plot the prediction with error bars representing the uncertainty in the prediction from the emulator and overlay the true values. We do this for each LHC in the full design, leaving that LHC out, refitting the emulator using the rest of the design, then plotting the predictions and truth against the fitted values or against each parameter in **x**. These plots we term LOLHO diagnostic plots. They allow us to check the consistency of our uncertainty specification, as in the leave one out situation, by seeing if enough/too many points are within the error bars, as well as finding any systematic sources of prediction error in certain locations of the parameter space that we may correct for, perhaps by adding further terms to *h*(**x**), or otherwise.

We produce *k* LOLHO plots, one for each of the different sub-hypercubes, as part of diagnostic checking and emulator validation, before finally using the full LHC to construct our emulator if we are happy with the diagnostics. We demonstrate the use of LOLHO plots in Section 11. Note that we should still perform some other diagnostic checks on our final model (Bastos & O'Hagan, 2009).

## 5. K-extended LHC design of a climate model ensemble

### 5.1. The model and its parameter space

**Figure 5:**
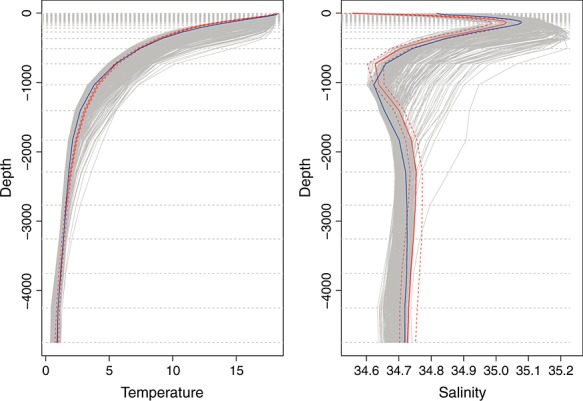
Global mean temperature and salinity depth profiles for the Nucleus for European Modelling of the Ocean ensemble (grey lines) with the standard setting of the parameters (blue depth profile) and the observed profiles (with uncertainty) the red solid and dashed lines.

We are interested in finding settings of the NEMO ocean model (Madec, 2008) that remove certain biases currently present in the model versions being explored by various projects concerned with the model. NEMO is an ocean model that takes, as inputs, atmospheric forcing files and settings of parameters to control sub-gridscale mixing. Figure 5 shows the output global mean temperature and salinity depth profiles for the current standard 2^*o*^ version of the model (blue line) compared with the observed temperature and salinity (red line, with dashed lines representing the observation uncertainty). The grey lines represent runs at the alternative parameter settings described in this section.

Following the approach of Williamson *et al.* (2014), where versions of the HadCM3 climate model (Gordon *et al.*, 2000) were found with dramatically improved ocean circulations, the ultimate goal will be to history match the 2^*o*^ version we have over several experiments wherein which the ocean model is run at parameter settings chosen from within a region of parameter space that is Not Ruled Out Yet by comparisons with observations. Emulators are built using these runs,and are then used to further reduce Not Ruled Out Yet space. History matching is an established statistical methodology for focussing a search for informative models through successive waves of comparison with observations. Craig *et al.* (1996) first applied it to oil reservoir models, and it has also been applied to Galaxy formation models (Vernon *et al.*, 2010) and climate models (Edwards *et al.*, 2011; Williamson *et al.*, 2013).

In all but the first wave, the parameter space will not be transformable to the unit hypercube (and may not even be simply connected depending on the constraints imposed by the observations), so special designs will be required to fill these spaces (for example, Williamson and Vernon, 2014). However, the initial parameter space, containing one switch variable with two settings and 20 continuous parameters defined on a hypercube with the ranges for each input elicited by the author from the developer of the code, Gurvan Madec, must be explored somehow and our preference is for a LHC design. As has been common in many applications we have worked on, very little is known about how the model will respond to the sort of large changes to the parameters that are possible in an elicited design space. Indeed, discovering how this response surface behaves is of great interest to the modellers involved.

The model is extremely computationally expensive, taking approximately 7.5h to complete 30years of model time on the UK supercomputer ARCHER. Running expensive climate models on supercomputers lends itself well to batch design. During the same 7.5h, we can run hundreds of parallel simulations. However, the pre-processing and post-processing of this information require human effort; hence, the modellers prefer to run in relatively large batches. We allocated 400 runs to this initial exploration of parameter space.

### 5.2. Experimental design

In order to quantify the major sources of uncertainty, it will be important to quantify uncertainty due to model parameters and due to perturbations to the initial conditions. Normally called ‘internal variability’ by climate modellers, this initial condition uncertainty will also depend on the model parameters. Much of this variability will be driven by the variability in the atmospheric forcing we impose upon the model and how it interacts with the ocean physics. There are two main options regarding the atmospheric forcing. We can force the model using observations taken over a 30-year period, or we can apply a constant forcing using a climatology. Initially, our collaborators prefer to apply climatological forcing to enable them to better understand the parametric response and to assist rapid convergence to equilibrium from rest. We run the model for 180 model years under climatological forcing, resubmitting the ensemble every 30years, and perform our analysis on the results.

At this point, it makes little sense to waste a large amount of computing resource and storage varying the initial conditions under climatological forcing, as the internal variability will be damped by the constant atmosphere. However, following spin up and observation of the parametric effect, we can continue each run applying the observed atmosphere. When the oceanographers decide to run these experiments then, at this point, initial condition uncertainty will become important, and we may want to use some of our computing resource to vary the initial conditions. To ensure that we can do this effectively when the time comes, we create our 400-member ensemble using a 25-extended 16 point LHC generated using the algorithm of Section 2. When we then perturb the initial conditions, we can choose one of these LHCs and select seven initial condition perturbations for each member. This would increase the ensemble size to 512, which is the largest size we can run without doubling the number of processors that need to be reserved on the supercomputer. This breakdown of our budget was chosen so that we felt we would have sufficient repeats to approximate the internal variability, and so that our initial condition ensemble was sufficiently space filling in order to us to capture any parameter dependencies in the initial condition uncertainty.

However, this particular breakdown of the design is not only useful if, in the future, we intend to explore the response to changes in the initial conditions. It allows us to build emulators with LOLHO diagnostics as described in Section 4.2. There were two settings of the only switch in the model. For the first 16-member LHC, which is earmarked for initial condition perturbations if the modellers decide to run those experiments, we select eight runs at each setting of the switch and configured them in order to minimize the maximum absolute correlation between the switch vector and any continuous input in the design. We then selected four sub-LHCs at random and fixed the switch at its first setting and four more at the other. This allows us to model differences between emulators developed for each switch setting if it becomes apparent that these are substantial. The remaining 16 LHCs were each randomly assigned eight values at each of the two settings. The configurations of all of the 25 switch vectors were then fixed; however, in order to minimize any correlation we may have introduced into the system, we searched through 1000 permutations of the last 24 vectors (preserving the order within each vector) for the lowest maximum absolute correlation between the switch vector and each continuous parameter vector in the large design. We note that with such a small sample size compared with the number of possible permutations, this configuration of switch settings is unlikely to be optimal with respect to, say, *ρ*^2^. Optimally configuring k-extended LHCs with switch variables would be an interesting topic of further investigation.

### 5.3. Emulating the ocean model

We will emulate the global mean sea surface temperature over the last 30years of the model output. Sea surface temperature is the ocean quantity for which we have the most complete real world data, so it is logical as part of our wider goal of history matching to begin with this output. Our emulator takes the form of model (4). We only provide brief details of the statistical modelling here, as neither emulation techniques, nor the emulation of this ocean general circulation model are the main topic of this paper. We report only the details required in order to present novel emulator diagnostics based on having a k-extended LHC design. Results of history matching ORCA2 to sea surface temperature and to other variables will be presented in another paper.

We begin the emulation process by searching for the regressors in the vector *h*(*x*) of Equation (4) using a forwards and backwards stepwise selection method, discussed in the appendix of Williamson *et al.* 2013). We then construct a Gaussian process emulator using the technology described by Haylock & O'Hagan (1996) and Kennedy & O'Hagan (2001), but adding a nugget term (so fitting model ((2), Andiranakis & Challenor, 2012). The covariance function for the Gaussian process was a power exponential form with power 1.9, as opposed to the usual fully Gaussian function with power 2, as it has been argued that this is often too smooth (Bayarri *et al.*, 2007).

We use the reference prior for the mean and variance parameters of the emulator as in Haylock & O'Hagan (1996), but specify a prior distribution for the correlation lengths and the nugget term (the latter is actually a parameter specifying the proportion of the residual variation (from the regression surface) that is uncorrelated ‘noise’). Our prior for the nugget is Beta(3.8,1.7), with the beta parameters selected by elicitation using the MATCH elicitation tool (Morris *et al.*, 2014). To specify a prior for the correlation parameters, we use the half-length correlation idea, which forms the prior question as one of thinking about a prior for the correlation between *ε*(*x*_*1*_) and *ε*(*x*_*2*_) where *x*_*1*_ and *x*_*2*_ are equal for all but the parameter in question, and where the distance between *x*_*1*_ and *x*_*2*_ is equal to half of the range of the parameter in question. We use a Beta prior for the half-length correlations of each parameter and elicit the parameters using the MATCH tool so that the prior for each half length correlation was Beta(2.9,5). This prior form allows us to derive our priors for the actual correlation parameters (see Williamson & Blaker, 2014, for more details).

To avoid running a long Markov Chain Monte Carlo whenever we want to evaluate the emulator, we fix the correlation and nugget parameters after conditioning on the ensemble, as suggested by Kennedy & O'Hagan (2001). We choose to fix these at their maximum a posteriori (MAP) estimates instead of maximum likelihood estimates, as these account for our prior modelling. MAP estimates are obtained using simulated annealing. We generate LOLHO plots by leaving out each of the 25 16-member sub-LHCs that make up our k-extended LHC design, in turn, and refitting the emulator to the remaining ensemble. Each time, we use the same *h*(*x*) and correlation parameters and recondition the prior with the reduced ensemble.

### 5.4. LOLHO diagnostics

**Figure 6:**
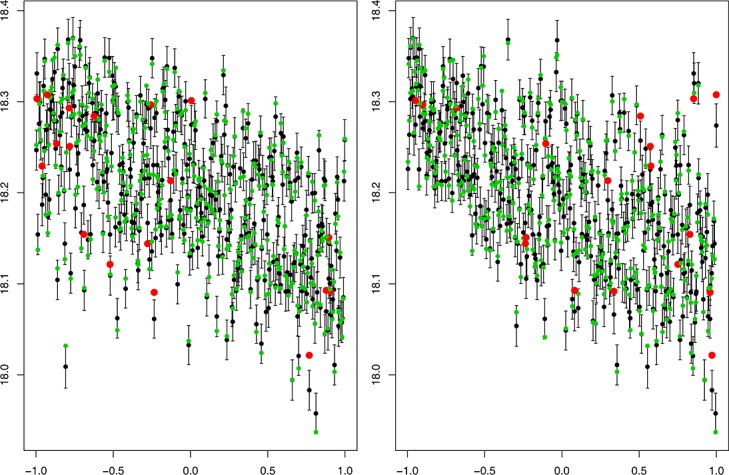
Traditional Leave One Out plots against the coefficient of Langmuir cells (left) and a coefficient controlling vertical eddies (right). The predictions and two standard deviation prediction intervals for the left out points are in black. The true values are in either green, if they are within two standard deviations of the prediction, or red otherwise

Figure 6 presents the traditional leave one out diagnostic for two of the model parameters: the Langmuir cells coefficient in the vertical mixing scheme (left panel) and a coefficient controlling the vertical behaviour of eddies (right panel). Each point represents an ensemble member that is left out of the ensemble, whilst the emulator is refitted using the same mean function and conditioned on the MAP estimates for the correlation lengths and nugget. The black points and error bars represent the emulator prediction and a two standard deviation prediction interval. The true (left out) values are then plotted in either green, if they are within two standard deviations of the prediction, or red (and larger) otherwise.

**Figure 7:**
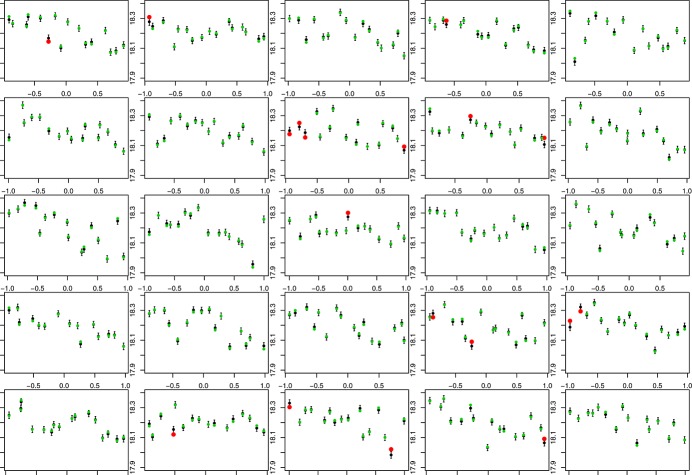
Leave One Latin Hypercube Out plots for each of the 25 16-member sub-Latin Hypercubes that made up our ocean model design. Each panel is constructed by removing a sub-Latin Hypercube from the design, refitting our emulator using the same basis function and correlation parameters and predicting the model output. The predictions and two standard deviation prediction intervals are in black. The true values are in either green, if they are within two standard deviations of the prediction, or red otherwise. The x-axis in each plot is the coefficient for Langmuir cells in the vertical mixing scheme.

**Figure 8:**
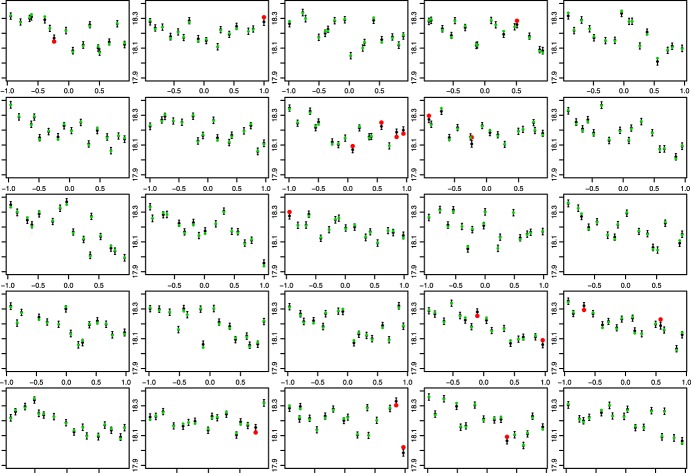
Leave One Latin Hypercube Out plots against a coefficient controlling vertical eddies, for each of the 25 16-member sub-Latin Hypercubes that made up our ocean model design.

The plots indicate that the emulator represents the model well. The prediction intervals are approximately 95% so we should expect around 20 points not to be within the uncertainty bounds, and we see 18. Additionally, we would be concerned if any of the red points were very far from the error bars, indicating a mis-specification of the variance across parameter space. We do not see any such points. Although these are useful plots, the LOLHO plots we present in Figures 7 and 8 offer further diagnostic insight. Each panel represents a left out LHC where at this time, the entire LHC is left out and all points predicted with the refitted emulator. Figure 7 plots each left out LHC against the Langmuir cells coefficient in the vertical mixing scheme, and Figure 8 plots each LHC against a coefficient controlling the vertical behaviour of eddies. In both figures, from the left, the first four panels depict LOLHO plots for the four hypercubes with the switch exclusively in its first setting, the next four in its second setting, and the remaining panels represent a balanced design in the switches.

**Figure 9:**
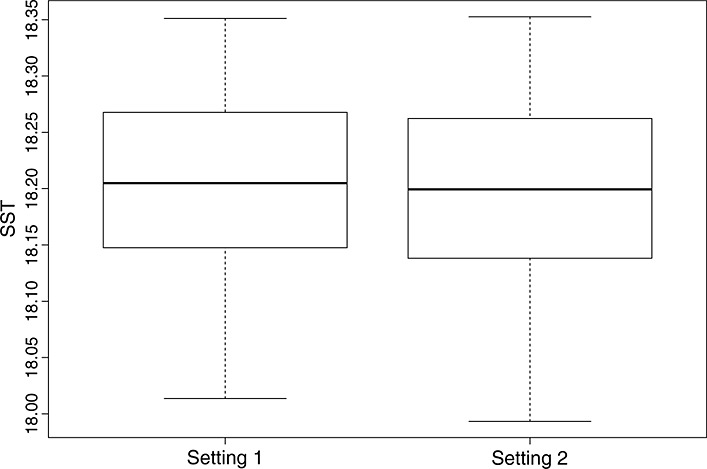
Boxplots illustrating the differences between emulator predictions for 100 new points in the continuous parameter space for each setting of the model switch.

Broadly, the plots indicate that there is little difference in our predictive capabilities for any setting of the switches. Figure 9 shows box plots for 100 predictions of the emulator at each switch setting, 100 alternative parameter choices, indicating that there is very little difference between the model output for the different switch settings in the full parameter space.

The breakdown of the diagnostic into distinct LHCs allows us to assess whether there are any areas of parameter space that do not validate well. For example, if the emulator were a good representation of our uncertainty, then the number of failures (red dots) in any individual panel, should be approximately binomial(16,0.05). Hence, we can compute the probability of seeing more than one failure in a given LHC (approximately 0.19) and the probability that we see more than three failures in one LHC (as we do in the eighth panel). Having five LHCs out of 25 with more than one failure is consistent with our uncertainty specification. The probability of more than three failures is 0.007; hence, we might view the eighth left out LHC as unusual and look at it in more detail.

We might start by seeing how unusual this result is. Suppose we view our LOLHO diagnostic as a sample from a binomial(25,0.007) distribution with success defined to be viewing more than three non-validating points in any plot. Then, the chance of having observed at least one such plot is 0.16, which is not particularly rare, although perhaps merits some investigation. Comparing the relevant panel in Figures 7 and 8, we can see that three of the failures are in the same corner of parameter space, where eddy mixing is large and the coefficient for Langmuir cells is small. Exploring some of the other panels, we see that this pattern is repeated (e.g. 2nd, 19th, 22nd and 23rd panels). The diagnostic, hence, suggests that our emulator may require more uncertainty in this corner of parameter space.

Whether we attempt to build this into a refined statistical model or not will depend on the purpose of the emulator. For example, if history matching reveals that this corner of parameter space leads to output that is far away from the observations, we may be happy with our current level of accuracy as we will cut this region of parameter space out anyway. Similarly, if this corner is always contained in the Not Ruled Out Yet region, again, we would be happy to take this emulator forward because our next design will be able to increase our density of model runs in this region and we will be able to build a new, accurate emulator anyway. If we are calibrating, and this region of parameter space appears to produce predictions that are close to observations, we might seriously consider building a more accurate emulator for the given region of parameter space as our emulators here will be influential for our calibrated predictions.

## 6. Discussion

We have presented a class of exploratory ensemble designs based on extending an algorithm for addition to existing designs first introduced by Sallaberry *et al.* (2008) that we call *k*-extended LHCs. They are composed of *k* smaller, equally sized, LHCs each added sequentially so that the composite design at each stage is ‘optimal’. Although an analyst may choose any properties to optimise using our algorithm, we also provide a criterion and algorithm for finding what we term ‘orthogonal-maximin k-extended LHCs’. K-extended LHCs are different from nested LHCs (Qian, 2009) in that, although each sub-cube may be thought of as nested in the overall design, the entire large design is composed of the union of such smaller LHCs. They can be thought of as a type of sliced LHC (Qian, 2012), LHCs that are entirely composed of smaller LHCs, with a construction that is focussed on optimising the composite designs of size *n*,2*n*,…,*kn*, sequentially.

We have argued for their use in applications such as those involving experiments with climate models, where the chaotic behaviour of the processes in the model means that the output varies with the initial conditions at which the model is run, so that some part of the design must be devoted to quantifying this uncertainty and its relationship to the model parameters. A particular advantage of k-extended LHCs in these applications is that we may optimise a single sub-LHC to be used for modelling initial condition uncertainty throughout the parameter space. Given an optimal configuration of the first sub-LHC, further LHCs are added to the design so that the composite design is optimal subject to the constraints imposed by the previous design. This property will be particularly useful in situations where the experiments are queued on supercomputers and when there is a chance, for whatever reason, that the whole queue may not complete before a deadline.

We described a novel emulator diagnostic called LOLHO plot, based on extending ‘leave one out’ methods. This diagnostic can be used for emulators built using k-extended and sliced LHCs. We provided a k-extended LHC design for a real ocean model, NEMO, that must be run on a supercomputer and showed the results of our emulation and LOLHO diagnostics for the largest ensemble of runs of NEMO we are aware of, run using a k-extended LHC design. Although storage and allocated run-time are factors affecting how such experiments are managed, often one of the biggest overheads is the time and experience of the modeller required to submit and manage the ensemble. If the storage and run time are available, a large ensemble takes the same amount of man hours to manage as a small one, so that the preference is often for fewer experiments (to the extent that this is possible whilst meeting the experimental goals) with larger ensembles at each step.

At the early stage of such a project, when little or nothing is known about how the model will respond to parameter changes, but where you may have been asked to use a large portion of your run budget, a design such as the one advocated here, where multiple sources of uncertainty can be addressed within one large LHC, is ideal. Although constructing a more involved LHC design, such as an orthogonal-maximin k-extended LHC (or even a sliced LHC) might be requiring more effort and personal computer time than using existing code to construct a more traditional maximin LHC, the computer time and effort will pale into comparison when compared with the cost (both in time and money) of the supercomputer experiment itself.

Space filling designs, such as LHCs, are only useful up to a point. Following an exploratory first experiment, it will be clear, after emulation of the computer model, that certain regions of parameter space are irrelevant to the analysis. For example, in a climate model, we may have an ice planet or no polar sea ice in winter, so that the model is a long way from the real world (and our uncertainty in our emulator predictions effectively means that we are sure that this is the case). Perhaps, the most interesting avenue for future research in the design of experiments will be in designs that aim to ‘fill’ the remaining space, however complex its shape may be, in some optimal way. Dragulic *et al.* (2012) began to think about this problem for parameter spaces with known constraints on the input variables. In many applications, however, all we have is a membership rule for parameter space with potentially very complex, unconnected shapes. Optimal designs in such spaces would have wide applicability for multi-wave computer experiments.

## APPENDIX A. DERIVING BOUNDS FOR THE COVERAGE STATISTIC

We take the same approach as Joseph & Hung (2008) in deriving the bounds for *φ*_*p*_ in Section 6. Joseph & Hung (2008)first prove two lemmas and use these to derive bounds. The first of these applies directly to designs generated during k-extension unchanged, so we simply state it as a result and refer interested readers to Joseph & Hung (2008) for the proof.

*Result* 1 Consider a set of positive values *d*_*j*,1_,*d*_*j*,2_,…,*d*_*j*,*l*_ and write *d*_*j*,(1)_≤·≤*d*_*j*,(*l*)_ for *j* = 1,…,*m*. Then,

The second of the required lemmas establishes the value of , the average inter-site distance in our design. However, because at Step 2 of the k-extension algorithm for any value of *c* we do not have an integer LHC, but rather a composite of *c**n* × *m* integer LHCs, we must state and prove a new result.

*Lemma* 1 For any value *c* at Step 2 of the k-extension algorithm, the integer representation of the current design and proposed extension, *M*^*c*^, has average inter-site distance

*Proof*. With our definition of *d*_*i*_, we have

However, every column of *M*^*c*^ contains *c* permutations of the integers {1,…,*n*}, so

Assessing a column with *c* 1s then *c* 2s and so on, ending with *c**n*s, we can write

and the result follows. □

The lower bound  is established on page 184 of Joseph & Hung (2008) as they show that using Lagrange multipliers to minimise  subject to  gives the optimal solution when . For the upper bound, because we have  for , by result 1

where now, for each column of the design, the inter-site distances are ordered in the same way by the right-most expression. For given *n* and *c*, each column will have  inter-site distances of 1/*k* and then, for each *h*∈{1,…,(*n* − 1)}, there will be *c*^2^(*n* − *h*) inter-site distances of *h*. Hence,

## SUPPORTING INFORMATION

Supporting info item

## References

[b1] Ai M, Jiang M, Li K (2013). Construction of sliced space-filling designs based on balanced sliced orthogonal arrays. Statistica Sinca.

[b2] Andiranakis I, Challenor PG (2012). The effect of the nugget on Gaussian process emulators of computer models. Computational statistics and data analysis.

[b3] Bastos LS, O'Hagan A (2009). Diagnostics for Gaussian process emulators. Technometrics.

[b4] Bayarri MJ, Berger JO, Cafeo J, Garcia-Donato G, Liu F, Palomo J, Parthasarathy RJ, Paulo R, Sacks J, Walsh D (2007). Computer model validation with functional output. Annals of Statistics.

[b5] Challenor P (2011). Designing a computer experiment that involves switches. Journal of Statistical Theory and Practice.

[b6] Craig PS, Goldstein M, Seheult AH, Smith JA, Bernado JM, Berger JO, Dawid AP, Smith AFM (1996). Bayes linear strategies for matching Hydrocarbon reservoir history. Bayesian Statistics 5.

[b7] Craig PS, Goldstein MCRJ, Seheult AH (2001). Bayesian forecasting for complex systems using computer simulators. Journal of the American Statistical Association.

[b8] Deser C, Phillips A, Bourdette V, Tang H (2012). Uncertainty in climate change projections: the role of internal variability. Climate Dynamics.

[b9] Dragulic D, Santner TJ, Dean AM (2012). Noncollapsing space-filling designs for bounded nonrectangular regions. Technometrics.

[b10] Edwards NR, Cameron D, Rougier JC (2011). Precalibrating an intermediate complexity climate model. Climate Dynamics.

[b11] Fang KT, Li R, Sudjianto A (2005). Design and Modeling for Computer Experiments.

[b12] Gordon C, Cooper C, Senior CA, Banks H, Gregory JM, Johns TC, Mitchell JFB, Wood RA (2000). The simulation of SST, sea ice extents and ocean heat transports in a version of the Hadley Centre coupled model without flux adjustments. Climate Dynamics.

[b13] Gramacy RB, Lee HKH, Bernardo JM, Bayarri MJ, Berger JO, Dawid AP, Heckerman D, Smith AFM, West M (2011). Optimization under unknown constraints. Bayesian Statistics.

[b14] Gu L, Yang JF (2013). Construction of nearly orthogonal Latin Hypercube designs. Metrika.

[b15] Haylock R, O'Hagan A, Bernado JM, Berger JO, Dawid AP, Smith AFM (1996). On inference for outputs of computationally expensive algorithms with uncertainty on the inputs. Bayesian Statistics 5.

[b16] Huang H, Yang JF, Liu MQ (2014). Construction of sliced (nearly) orthogonal Latin hypercube designs. Journal of Complexity.

[b17] Iman RL, Conover WJ (1982). A distribution free approach to introducing rank correlation between variables. Communications in Statistics-Simulation and Computation B.

[b18] Joseph R, Hung Y (2008). Orthogonal-maximin Latin Hypercube designs. Statistica Sinca.

[b19] Kennedy MC, O'Hagan A (2001). Bayesian calibration of computer models. Journal of the Royal Statistical Society Series B.

[b20] Lee LA, Carslaw KS, Pringle KJ, Mann GW, Spracklen DV (2011). Emulation of a complex global aerosol model to quantify sensitivity to uncertain parameters. Atmospheric Chemistry and Physics.

[b21] Loeppky JL, Moore LM, Williams BJ (2009). Batch sequential designs for computer experiments. Journal of Statistical Planning and Inference.

[b22] Madec G (2008).

[b23] McKay M, Beckman R, Conover W (1979). A comparison of three methods for selecting values of input variables in the analysis of output from a computer code. Technometrics.

[b24] Morris MD, Mitchell TJ (1995). Exploratory designs for computational experiments. Journal of Statistical Planning and Inference.

[b25] Morris DE, Oakley JE, Crowe JA (2014). A web-based tool for eliciting probability distributions from experts. Environmental Modelling and Software.

[b26] Oakley JE, O'Hagan A (2004). Probabilistic sensitivity analysis of complex models: a Bayesian approach. Journal of the Royal Statistical Society Series B.

[b27] Qian PZG (2009). Nested latin hypercube designs. Biometrika.

[b28] Qian PZG (2012). Sliced latin hypercube designs. Journal of the American Statistical Association.

[b29] Rougier JC (2013). Intractable and unsolved?: some thoughts on statistical data assimilation with uncertain static parameters. Philosophical Transactions of the Royal Society A.

[b30] Sacks J, Welch WJ, Mitchell TJ, Wynn HP (1989). Design and analysis of computer experiments. Statistical Science.

[b31] Sallaberry CJ, Helton JC, Hora SC (2008). Extension of Latin Hypercube samples with correlated variables. Reliability Engineering and System Safety.

[b32] Santner TJ, Williams BJ, Notz WI (2003). The Design and Analysis of Computer Experiments.

[b33] Sun FS, Liu MQ, Lin DKJ (2009). Construction of orthogonal Latin Hypercube designs. Biometrika.

[b34] Tang B (1993). Orthogonal array-based Latin Hypercubes. Journal of the American Statistical Association.

[b35] Vernon I, Goldstein M, Bower RG (2010). Galaxy formation: a Bayesian uncertainty analysis. Bayesian Analysis.

[b36] Vernon I, Goldstein M (2010). http://www.mucm.ac.uk/Pages/Downloads/Technical%20Reports/10-10%202.1.14\%20IV.pdf.

[b37] Vernon I, Goldstein M (2014).

[b38] Williamson D, Goldstein M (2012). Bayesian policy support for adaptive strategies using computer models for complex physical systems. Journal of the Operational Research Society.

[b39] Williamson D, Goldstein M, Blaker AT (2012). Fast linked analyses, for scenario-based hierarchies. Journal of the Royal Statistical Society Series C.

[b40] Williamson D, Goldstein M, Allison L, Blaker A, Challenor P, Jackson L, Yamazaki K (2013). History matching for exploring and reducing climate model parameter space using observations and a large perturbed physics ensemble. Climate Dynamics.

[b41] Williamson D, Blaker AT (2014). Evolving Bayesian emulators for structurally chaotic time series with application to large climate models. SIAM/ASA Journal of Uncertainty Quantification.

[b42] Williamson D, Vernon IR (2014).

[b43] Williamson D, Blaker AT, Arnfield M, Hampton C, Salter J (2014). Identifying and removing structural biases in climate models with history matching. Climate Dynamics.

[b44] Yin Y, Lin DKJ, Liu MQ (2014). Sliced Latin Hypercube designs via orthogonal arrays. Journal of Statistical Planning and Inference.

